# Medial Temporal Lobe Subregional Atrophy in Aging and Alzheimer's Disease: A Longitudinal Study

**DOI:** 10.3389/fnagi.2021.750154

**Published:** 2021-10-15

**Authors:** Léa Chauveau, Elizabeth Kuhn, Cassandre Palix, Francesca Felisatti, Valentin Ourry, Vincent de La Sayette, Gaël Chételat, Robin de Flores

**Affiliations:** ^1^U1237 PhIND, Inserm, Caen-Normandie University, GIP Cyceron, Caen, France; ^2^U1077 NIMH, Inserm, Caen-Normandie University, École Pratique des Hautes Études, Caen, France

**Keywords:** medial temporal lobe (MTL), Alzheimer's disease, aging, episodic memory, structural magnetic resonance imaging, mild cognitive impairment, hippocampus

## Abstract

Medial temporal lobe (MTL) atrophy is a key feature of Alzheimer's disease (AD), however, it also occurs in typical aging. To enhance the clinical utility of this biomarker, we need to better understand the differential effects of age and AD by encompassing the full AD-continuum from cognitively unimpaired (CU) to dementia, including all MTL subregions with up-to-date approaches and using longitudinal designs to assess atrophy more sensitively. Age-related trajectories were estimated using the best-fitted polynomials in 209 CU adults (aged 19–85). Changes related to AD were investigated among amyloid-negative (Aβ−) (*n* = 46) and amyloid-positive (Aβ+) (*n* = 14) CU, Aβ+ patients with mild cognitive impairment (MCI) (*n* = 33) and AD (*n* = 31). Nineteen MCI-to-AD converters were also compared with 34 non-converters. Relationships with cognitive functioning were evaluated in 63 Aβ+ MCI and AD patients. All participants were followed up to 47 months. MTL subregions, namely, the anterior and posterior hippocampus (aHPC/pHPC), entorhinal cortex (ERC), Brodmann areas (BA) 35 and 36 [as perirhinal cortex (PRC) substructures], and parahippocampal cortex (PHC), were segmented from a T1-weighted MRI using a new longitudinal pipeline (LASHiS). Statistical analyses were performed using mixed models. Adult lifespan models highlighted both linear (PRC, BA35, BA36, PHC) and nonlinear (HPC, aHPC, pHPC, ERC) trajectories. Group comparisons showed reduced baseline volumes and steeper volume declines over time for most of the MTL subregions in Aβ+ MCI and AD patients compared to Aβ− CU, but no differences between Aβ− and Aβ+ CU or between Aβ+ MCI and AD patients (except in ERC). Over time, MCI-to-AD converters exhibited a greater volume decline than non-converters in HPC, aHPC, and pHPC. Most of the MTL subregions were related to episodic memory performances but not to executive functioning or speed processing. Overall, these results emphasize the benefits of studying MTL subregions to distinguish age-related changes from AD. Interestingly, MTL subregions are unequally vulnerable to aging, and those displaying non-linear age-trajectories, while not damaged in preclinical AD (Aβ+ CU), were particularly affected from the prodromal stage (Aβ+ MCI). This volume decline in hippocampal substructures might also provide information regarding the conversion from MCI to AD-dementia. All together, these findings provide new insights into MTL alterations, which are crucial for AD-biomarkers definition.

## Introduction

The hippocampus (HPC), entorhinal cortex (ERC), perirhinal cortex (PRC), and parahippocampal cortex (PHC) are subregions of the medial temporal lobe (MTL) that are critical for several cognitive functions such as declarative memory and spatial navigation (Wixted and Squire, 2011; Raslau et al., 2015; Moscovitch et al., 2016; Baumann and Mattingley, 2021). Importantly, these subregions are altered early and severely in Alzheimer's disease (AD), as they are the primary target of neurofibrillary tangles (Braak and Braak, 1991, 1995; Schöll et al., 2016). In this context, hippocampal atrophy, assessed by structural magnetic resonance imaging (MRI), has been extensively studied in Alzheimer's research and is widely used as a biomarker to monitor *in-vivo* AD-related neurodegeneration (Frisoni et al., 2010; Pini et al., 2016). For example, a study focusing on the HPC reported an annual volume reduction of 1.9 ± 0.9% in patients with AD-dementia, and 1.3 ± 0.9% in patients with mild cognitive impairment (MCI) (Henneman et al., 2009). In addition, hippocampal volumetry might be a relevant tool to predict the conversion to AD-dementia in patients with MCI (Pennanen et al., 2004; Apostolova et al., 2006; Devanand et al., 2007; Henneman et al., 2009; Brueggen et al., 2015). As neuropathological changes are known to start several years before symptom onset, more and more studies have focused on the preclinical stage of AD—defined as amyloid-positive (Aβ+) cognitively unimpaired (CU) individuals (Jack et al., 2018)—(Miller et al., 2013; Fortea et al., 2014; Pegueroles et al., 2017; Pettigrew et al., 2017); findings are sparse with only a few studies showing medial temporal lobe subregional atrophy in Aβ+ compared to amyloid-negative (Aβ−) CU (Wolk et al., 2017; Xie et al., 2020), while others have found no difference (Xie et al., 2019).

Interestingly, MTL subregions are also affected in typical aging, and dissociating normal from early pathological changes is sometimes challenging (Fjell et al., 2014). For example, studies report a decline in HPC and ERC volumes with age (Schuff et al., 1999; Jernigan et al., 2001; Du et al., 2006), and trajectories appear to differ across MTL subregions with linear vs. non-linear atrophy over the whole lifespan (Ziegler et al., 2012; Fjell et al., 2013; de Flores et al., 2015; Daugherty et al., 2016; Hasan et al., 2016; Bussy et al., 2021). Thus, a better understanding of the effects of age on the MTL subregions is essential to define the best candidate to be used as an AD biomarker.

To date, most of the investigations on MTL structural alterations were performed using cross-sectional designs and were mainly limited to the HPC and ERC (Juottonen et al., 1998; Du et al., 2001, 2003; Pennanen et al., 2004; Barnes et al., 2009; Wisse et al., 2014; Pini et al., 2016). Given that MTL subregions are affected heterogeneously in AD (Braak and Braak, 1995; Krumm et al., 2016; Xie et al., 2020), longitudinal designs accounting for inter-individual variability and including usually neglected MTL subregions such as the PRC and PHC might enhance the clinical utility of these neurodegeneration biomarkers. In addition, automatic procedures recently emerged to segment MTL subregions from standard T1-weighted MRI scans (Pipitone et al., 2014; Iglesias et al., 2015; Xie et al., 2019), and appear promising in characterizing AD-related changes in clinical routines as MRI images are more accessible compared with other neuroimaging techniques which are more expensive and invasive.

In this context, this study aims to better characterize the structural alterations of MTL subregions in typical aging and across the whole Alzheimer's continuum. For this purpose, we took advantage of the longitudinal T1-weighted MRI data acquired from 209 CU participants covering the adult lifespan and 84 patients with MCI and AD. More precisely, we used a tailored methodology to (i) explore the age-related volume trajectories of MTL subregions over the adult lifespan, (ii) examine the differences in MTL subregional atrophy across the Alzheimer's continuum (encompassing Aβ− CU older adults [i.e., controls], Aβ+ CU older adults, Aβ+ MCI patients, and Aβ+ AD patients), (iii) compare MTL subregional atrophy between MCI-to-AD converters and non-converters patients, and finally, (iv) investigate the associations between the volume of MTL subregions and cognitive performances.

## Materials and Methods

### Participants

All participants in this study were enrolled in the *multi-modal neuroimaging in AD* study (IMAP+) (Caen, France) between the years 2008 and 2016, and some of them were included in previous publications from our lab (La Joie et al., 2013; de Flores et al., 2015; Perrotin et al., 2017). All were right-handed, had at least 7 years of education, and had no history of alcoholism, drug abuse, head trauma, or neurological/psychiatric disorder. The IMAP+ study was approved by a regional ethics committee (Comité de Protection des Personnes Nord-Ouest III) and was registered with ClinicalTrials.gov (number NCT01638949). All participants gave written informed consent to the study before the investigation.

Two hundred and nine CU adults from the ages 19–85 were recruited from the community using flyers and advertisements in local newspapers, and had normal cognitive performances according to age and education level, without memory complaints. Patients were recruited from a local memory clinic and then stratified by a senior neurologist and neuropsychologists according to standard clinical criteria. Thirty-one patients with AD fulfilled the standard National Institute of Neurological and Communicative Disorders and Stroke and the Alzheimer's Disease and Related Disorders Association (NINCDS-ADRDA) clinical criteria for mild to moderate probable AD (McKhann et al., 1984), and 53 patients with MCI satisfied Petersen's criteria (Petersen and Morris, 2005) at baseline. Among the 53 patients with MCI, 19 converted to AD-dementia (updated on October 23, 2020, post-study clinical follow-up = 20.7 ± 22.9 months).

Participants were followed up to 47 months with one to three neuroimaging/cognitive assessments (±18 months apart). Florbetapir-PET scans were available for each individual and Aβ−positivity was defined using a global neocortical standardized uptake value ratio (SUVr) measure. The positivity threshold was calculated as the 99.9th percentile of the distribution among 45 healthy individuals younger than 40, corresponding to a SUVr of 0.99. SUVr images were corrected for partial volume effect using the three-compartment method (Müller-Gärtner et al., 1992). Among the CU aged over 60, 46 were Aβ− and 14 were Aβ+, while 33 MCI and 31 AD patients were Aβ+. Of note, Aβ status was determined using scans acquired at baseline for the vast majority of our population. However, when the SUVr value was not available at baseline but values at following times were below the threshold, we considered the individual as Aβ− (*n* = 21). In addition, apolipoprotein E (*APOE*) genotype was available for 203 of the 209 CU, resulting in 53 ε4 allele carriers and 150 non-carriers.

The demographics of our samples are presented in Table 1.

**Table 1:** Demographics of the study sample.

	**Adult Lifespan**		**Alzheimer's continuum**			**MCI-to-AD**	
	**CU**	**CU Aβ−**	**CU Aβ+**	**MCI Aβ+**	**AD Aβ+**	**Non-converters**	**Converters**
Sex: n_female_/n_male_	111/98	26/20	8/6	13/20	13/18	15/19	6/13
Age (years)	46.9 ± 18.6 [19.5, 84.6]	69.3 ± 5.9 [60, 81.4]	73.8 ± 7.2^*^ [61.5, 84.6]	73.9 ± 7.2^**^ [59.9, 86.6]	67.3 ± 9.9 [51.7, 84.1]	73.7 ± 7.1 [58.8, 85.1]	72.5 ± 7.1 [60.7, 85.0]
Education (years)	13.4 ± 3.3 [7, 20]	12.3 ± 3.9 [7, 20]	11.3 ± 3.3 [7, 17]	11.6 ± 4 [6, 20]	10.9 ± 3.2 [6, 20]	10.6 ± 2.9 [6, 17]	12 ± 4.7 [7, 20]
*APOE4*^a^: n_carrier_/n_non−carrier_	53/150	–	–	–	–	–	–
Follow-up (months)	18.8 ± 11.2 [0, 47.2]	26.9 ± 11.9 [0, 41.2]	20.7 ± 12.5 [0, 39.8]	18.6 ± 13.1 [0, 40.7]	12.8 ± 9.1 [0, 22.6]	21.4 ± 15.5 [0, 43.9]	22.5 ± 12.9 [0, 37.1]
MMSE^b^	29.2 ± 0.9 [26, 30]	28.7 ± 1.2 [26, 30]	29.3 ± 0.7 [28, 30]	26.8 ± 1.9^***^ [22, 30]	20 ± 4.9^***^ [12, 29]	26.9 ± 1.9 [22, 30]	26.3 ± 1.8 [22, 30]
Post-study clinical follow-up (months)	–	–	–	–	–	16.3 ± 21.2 [0, 89.7]	28.3 ± 24.5 [0, 78]

*Continuous variables are indicated as follows: mean ± SD [minimum, maximum]*.

*Age, education, MMSE from baseline*.

*CU Aβ+, MCI Aβ+, and AD Aβ+ were compared with CU Aβ−; converters were compared with non-converters. Independent two-sample t-test (age), Wilcoxon rank-sum test (education and MMSE), and chi-squared test (sex) were used for statistical comparisons*.

*** *p < 0.001;*

** *p < 0.01;*

* *p < 0.05*.

a *Missing data for six participants*.

b *Missing data for two participants*.

*AD, patients who met McKhann criteria for probable Alzheimer's disease dementia; APOE, apolipoprotein E; CU, cognitively unimpaired participants; MCI, mild cognitive impairment; MMSE, mini-mental state examination*.

### MRI Data Acquisition

Participants were repeatedly scanned with a Philips Achieva 3T camera at the Cyceron Center (Caen, France). Longitudinal T1-weighted anatomical images were acquired using a three-dimensional (3D) fast-field echo sequence (3D-T1-FFE sagittal; SENSE factor =2; repetition time = 20 ms; echo time = 4.6 ms; flip angle = 10°; 180 slices with no gap; slice thickness = 1 mm; field of view = 256 × 256 mm^2^; in-plane resolution = 1 × 1 mm^2^).

### Automatic Segmentation of Medial Temporal Lobe Subregions

MTL subregions, including the anterior and the posterior hippocampus (aHPC and pHPC, respectively), the ERC, Brodmann areas (BA) 35 and 36—as PRC components –, and the PHC were automatically segmented using the *Automatic Segmentation of Hippocampal Subfields* (ASHS) software (Yushkevich et al., 2015; atlas: ashsT1_atlas_upennpmc_07202018; Supplementary Figure 1). This multi-atlas segmentation algorithm has the advantage of accounting for the dura mater confounds and MTL cortex anatomic variability (Xie et al., 2019). Participants with a single neuroimaging assessment (usually baseline) were directly segmented using the standard ASHS-T1 pipeline, while the *Longitudinal Automatic Segmentation of Hippocampal Subfields* (LASHiS) pipeline (Shaw et al., 2020) was used to segment images from participants with longitudinal follow-ups (two or three assessments). This method is directly based on the ASHS algorithm but additionally takes account of the longitudinal aspect of the data by building a single subject template, thus, reducing random errors in the labeling procedure. Rigorous quality control was performed by visually evaluating the segmentation of each subregion for all participants. Failed segmentations were manually edited when feasible (~30%, for aHPC or pHPC only) or were discarded. Note that for the same individual, some subregions were of adequate quality to be included in analyses while others were not, so the number of subjects for a given MTL subregion was not the same (Supplementary Tables 2–9).

The aHPC and pHPC volumes were summed to obtain the volume of the whole HPC, as well as the BA 35 and 36 volumes to estimate the PRC volume. Left and right volumes were averaged to limit the number of analyses. Raw bilateral volumes were then normalized by the total intracranial volume (TIV-calculated as the sum of the volumes of gray matter, white matter, and cerebrospinal fluid using SPM12) to account for inter individual variability in head size (normalized volume raw = volume 100/TIV), and then z-transformed to improve comparability between the subregions. For this transformation, we used (i) data from baseline CU adults under 40 for analyses performed in the context of typical aging and (ii) data from baseline Aβ− CU older adults for analyses performed in the context of pathological aging.

### Cognitive Functioning

Participants repeatedly underwent the same neuropsychological exams as described in detail previously (Chételat et al., 2005; Mevel et al., 2013). To obtain robust proxies of cognitive abilities and to minimize the issue of multiple statistical testing, we calculated composite scores reflecting executive functions, processing speed, and episodic memory. Composite scores were derived from individual scores z-transformed using baseline Aβ− CU older adults as the reference since cognition was only investigated in patients. We obtained the processing speed score from the duration of the Trail Making Test (TMT) (Reitan, 1958) part A and the Stroop (1935) color naming. The executive functions score was computed from a flexibility score calculated with the TMT ((part B – part A)/part A) and an inhibition score calculated with the Stroop [(interference – color naming)/color naming]. The episodic memory score averaged the free recalls of the Encoding, Storage, Retrieval (ESR) paradigm (Eustache et al., 1998), the adapted *Batterie d'efficience mentale* (BEM)-144 (Signoret, 1991), and the BEM figure. For all composite scores, higher values indicate better performances. Since some patients failed to complete all the tests, the sample size for a given cognitive domain varies (Supplementary Table 9).

### Statistical Analyses

All statistical analyses were performed using R (version 4.0.3; R Core Team, 2020). Longitudinal data were analyzed using linear mixed models (LMMs). These models are advantageous in handling within-subject observations, as well as missing data and unbalanced designs (e.g., irregular follow-ups as in the current study). LMMs were fitted to the data with restricted maximum likelihood, using the *lme4* package (Bates et al., 2015). Type III F-tests with Satterthwaite's method for degrees of freedom approximation was used to assess the significance of fixed effects. Effect size estimates of the LMMs were assessed using pseudo-R-squared (Nakagawa et al., 2017). Results were considered significant after Holm's family-wise error rate controlling procedure to account for multiple tests (*p* < 0.05) (Holm, 1979). Model quality was assessed using the *performances* package (Lüdecke et al., 2021), and visual inspection of the residual plots did not reveal any obvious deviations from homoscedasticity or normality.

#### Age-Related Volume Trajectories Over the Adult Lifespan

Relationships between age and MTL subregional volumes were estimated over the adult lifespan in CU participants aged from 19 to 85 years old. LMMs with polynomials of different orders for the age term were considered to best describe the age-related volume trajectories of MTL subregions. Models were tested from the simplest to the most complex. A model was kept as a candidate when the likelihood ratio test (i.e., testing *n* vs. *n-1* order-polynomial) was simultaneously significant (*p* < 0.05) and when all the coefficients were significant using *t*-statistic (*p* < 0.05). Candidate models were compared using the corrected Akaike Information Criterion (AICc) and the Bayesian Information Criterion (BIC) according to criteria (Burnham and Anderson, 2004). As an exception, LMMs were fitted with maximum likelihood for model comparison (Zuur et al., 2009). More details on the model selection are provided in the Supplementary Method. For the nonlinear relationships, the inflections in the smoothed curves were estimated from the place where a change in model estimates switches signs. Separate LMMs with each MTL subregion as dependent variable were computed. Fixed effect terms included *age* as an independent variable and *sex* and *education* as covariates. Intercepts for participants were entered as random effects. Differences related to sex, education level, or *APOE* ε4 gene in age trajectories were also investigated by adding the variable of interest (i.e., *sex, education*, or *apoe*) and its interaction with the *age* term as fixed effects.

#### Structural Alterations Across the Alzheimer's Continuum

Structural alterations of MTL subregions across the Alzheimer's continuum were investigated in 124 participants, pooling CU participants over 60 years of age [both Aβ− (*n* = 46) and Aβ+ (*n* = 14)], as well as Aβ+ MCI (*n* = 33) and AD (*n* = 31) patients. Baseline volume differences were assessed using analyses of covariance (ANCOVAs) with group (ie, Aβ− CU, Aβ+ CU, Aβ+ MCI, and Aβ+ AD) as predictors. To estimate the volume changes over time, LMMs with random intercept and slope per participant were then computed with *group, time*, and their interaction as fixed effects. In both ANCOVAs and LMMs, each MTL subregion was entered as a dependent variable in separate models. All models were controlled for *age, sex*, and *education*. To compare atrophy across groups, independent *t*-tests adjusted for multiple comparisons using Tukey honestly significant difference test were used as *post-hoc* comparing estimated marginal means with the *emmeans* package (Lenth et al., 2021).

#### Volume Comparisons Between Converters and Non-converters

MTL subregional atrophy was compared between 19 MCI-to-AD converters and 34 non-converters. Differences in baseline volumes were investigated using ANCOVAs, and differences in volume decreases using LMMs with by-participant random intercepts. Each MTL subregion was entered as a dependent variable in both types of models. The *group* (i.e., converters or non-converters) was included as predictor in ANCOVAs, and *group, time*, and their interaction were included as fixed effects in LMMs. In all models, *post-study clinical follow-up, age, sex*, and *education* were added as covariates.

#### Relationships Between Cognitive Functioning and Volume

Associations between the cognitive scores and MTL subregions volume were only investigated in Aβ+ MCI and AD patients to avoid ceiling effects and to get enough variance in the scores. Separate LMMs were conducted for each cognitive score (reflecting speed processing, executive functions, and episodic memory) as a dependent variable and for each MTL subregion as a predictor (i.e., 24 tests). Random effects included subject intercepts. All models were adjusted according to the *group, time, age, sex*, and *education*.

## Results

### Aging Differently Impairs MTL Subregions Over the Adult Lifespan

**Figure 1:**
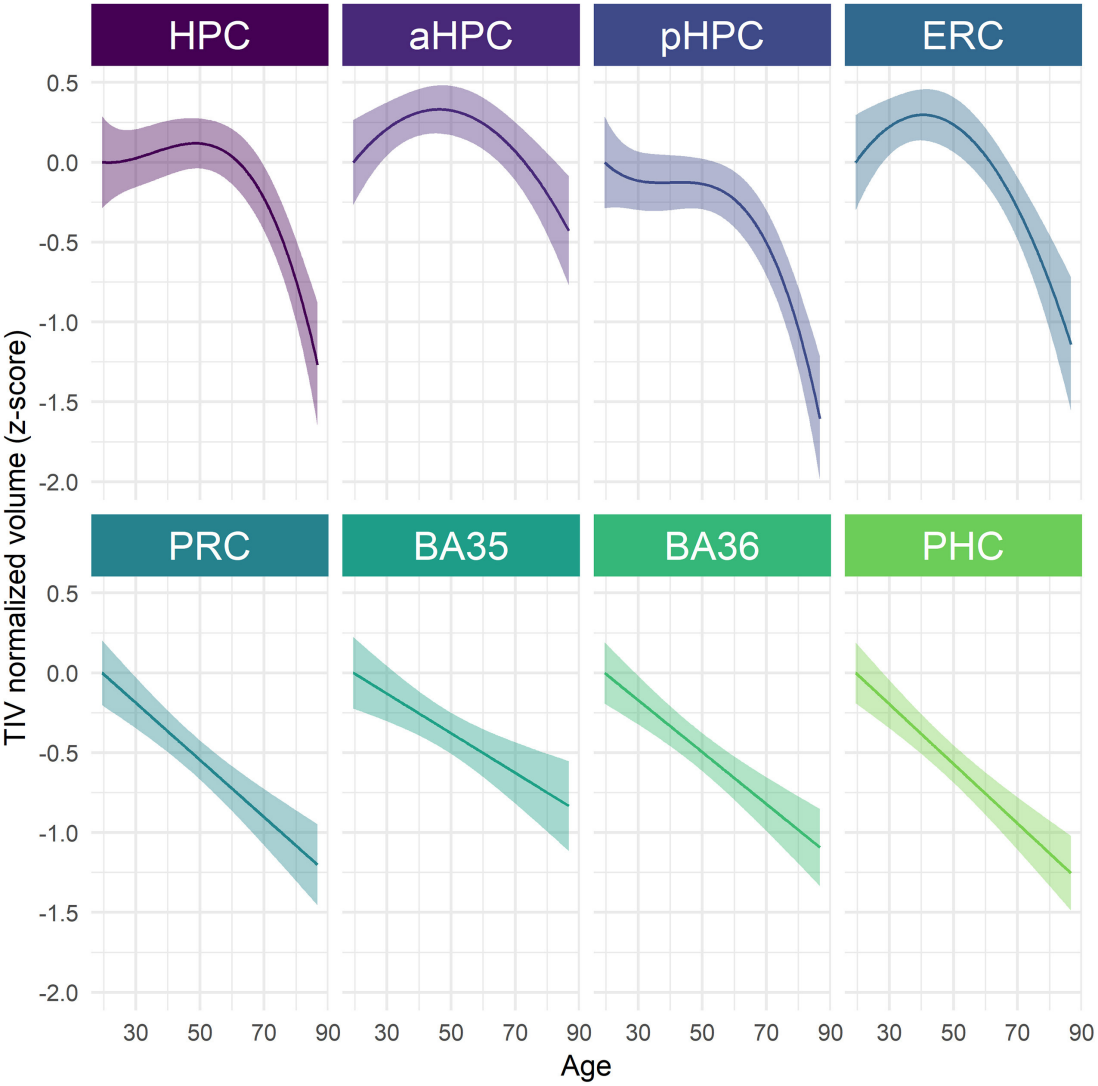
Age-related volume trajectories of MTL subregions over the adult lifespan. All subregions were significantly related to age, *p* < 0.001. Continuous line indicates model-derived estimates. The shaded area represents a 95% confidence interval. BA, Brodmann area; ERC, entorhinal cortex; HPC, hippocampus (a, anterior; p, posterior); PHC, parahippocampal cortex; PRC, perirhinal cortex.

Structural trajectories over the adult lifespan of all the considered MTL subregions are displayed in Figure 1. All MTL subregions volume were significantly related to age using the best-fitted models [HPC: *F*_(317.89)_ = 14.58, *p* < 0.001; aHPC: *F*_(344.43)_ = 8.05, *p* < 0.001; pHCP: *F*_(316.73)_ = 17.49, *p* < 0.001; ERC: *F*_(309.85)_ = 16.37, *p* < 0.001; PRC: *F*_(318.03)_ = 37.23, *p* < 0.001; BA35: *F*_(290.60)_ = 14.11, *p* < 0.001; BA36: *F*_(339.54)_ = 35.04, *p* < 0.001; PHC: *F*_(370.18)_ = 48.41, *p* < 0.001). Both linear and non-linear age-related trajectories were found. More precisely, the BA 35 and 36, PRC, and PHC volumes were linearly associated with age, a quadratic term better described this relationship between volume and age for the aHCP and ERC, and a cubic term better described this relationship for the HPC and pHPC (Supplementary Table 1). The estimation of inflection points suggested that the aHPC volume declined from the age of 47 and the ERC volume from the age of 41. The HPC volume approximately increased at the age of 22 and then decreased at 49. The pHPC volume exhibited stability from the ages 37–44 and then started to decline.

When investigating the sex-related differences, a significant main effect of *sex* was observed for pHPC [*F*_(202.88)_ = 7.94, *p* = 0.04] and PHC [*F*_(204.66)_ = 23.38, *p* < 0.001], suggesting that women have greater volumes than men (Supplementary Figure 2, Supplementary Table 2). No significant *age* × *sex* interaction was found, suggesting that age trajectories were comparable among men and women (Supplementary Table 2). With regard to differences related to *APOE4* or education level, results revealed neither an interaction effect with *age* nor a simple effect of the variable of interest (i.e., *apoe* or *education*) for none of all considered MTL subregions (Supplementary Tables 3, 4).

### AD Affects MTL Subregions More Severely in the Advanced Stages of the Continuum

**Figure 2:**
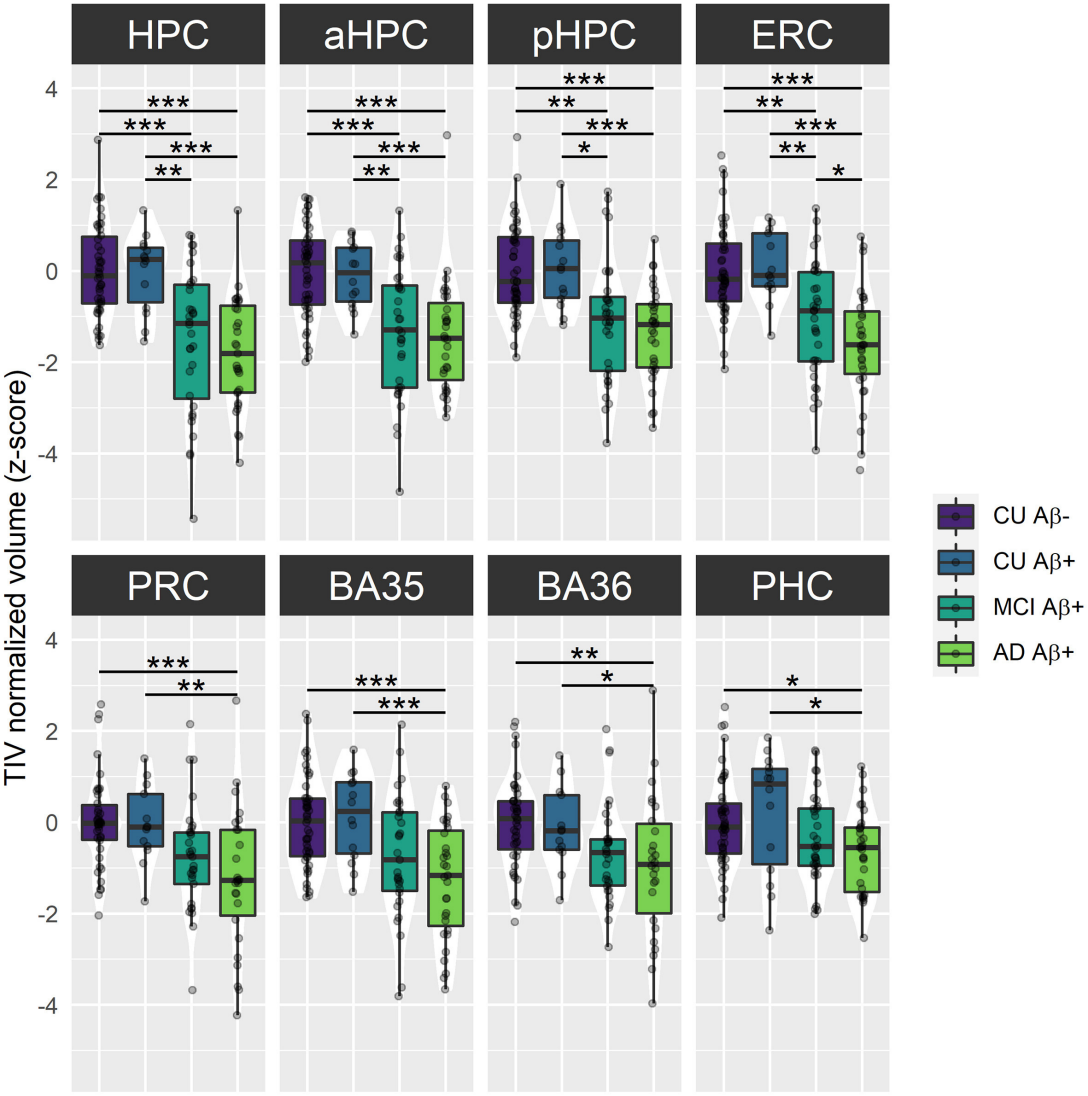
Group comparisons of MTL subregions baseline volume across the Alzheimer's continuum. Boxplots display the median values and distribution of z-transformed TIV normalized volumes at baseline in each group. ***p < 0.001; **p < 0.01; *p < 0.05, as results of Tukey adjusted *post-hoc t*-tests. AD, Alzheimer's dementia; BA, Brodmann area; CU, cognitively unimpaired; ERC, entorhinal cortex; HPC, hippocampus (a, anterior; p, posterior); MCI, mild cognitive impairment; PHC, parahippocampal cortex; PRC, perirhinal cortex. The corresponding statistics are depicted in Supplementary Table 5.

Cross-sectional analyses revealed significant effects of *group* on baseline volume for all MTL subregions [HPC: *F*_(3)_ = 19.32, *p* < 0.001; aHPC: *F*_(3)_ = 14.25, *p* < 0.001; pHPC: *F*_(3)_ = 14.04, *p* < 0.001; ERC: *F*_(3)_ = 17.36, *p* < 0.001; PRC: *F*_(3)_ = 7.09, *p* < 0.001; BA35: *F*_(3)_ = 9.16, *p* < 0.001; BA36: *F*_(3)_ = 4.56, *p* = 0.005; PHC: *F*_(3)_ = 3.96, *p* = 0.01]. As illustrated in Figure 2, no significant difference between Aβ− and Aβ+ CU was observed. Aβ+ MCI patients showed significantly smaller HPC, aHPC, pHPC, and ERC volumes compared to Aβ− CU, and to Aβ+ CU. Aβ+ AD patients showed significantly smaller volumes in all MTL subregions compared to Aβ− CU, and to Aβ+ CU. Lastly, Abβ+ AD showed significantly smaller ERC volumes compared to Abβ+ MCI patients. The corresponding statistics are depicted in Supplementary Table 5.

**Figure 3:**
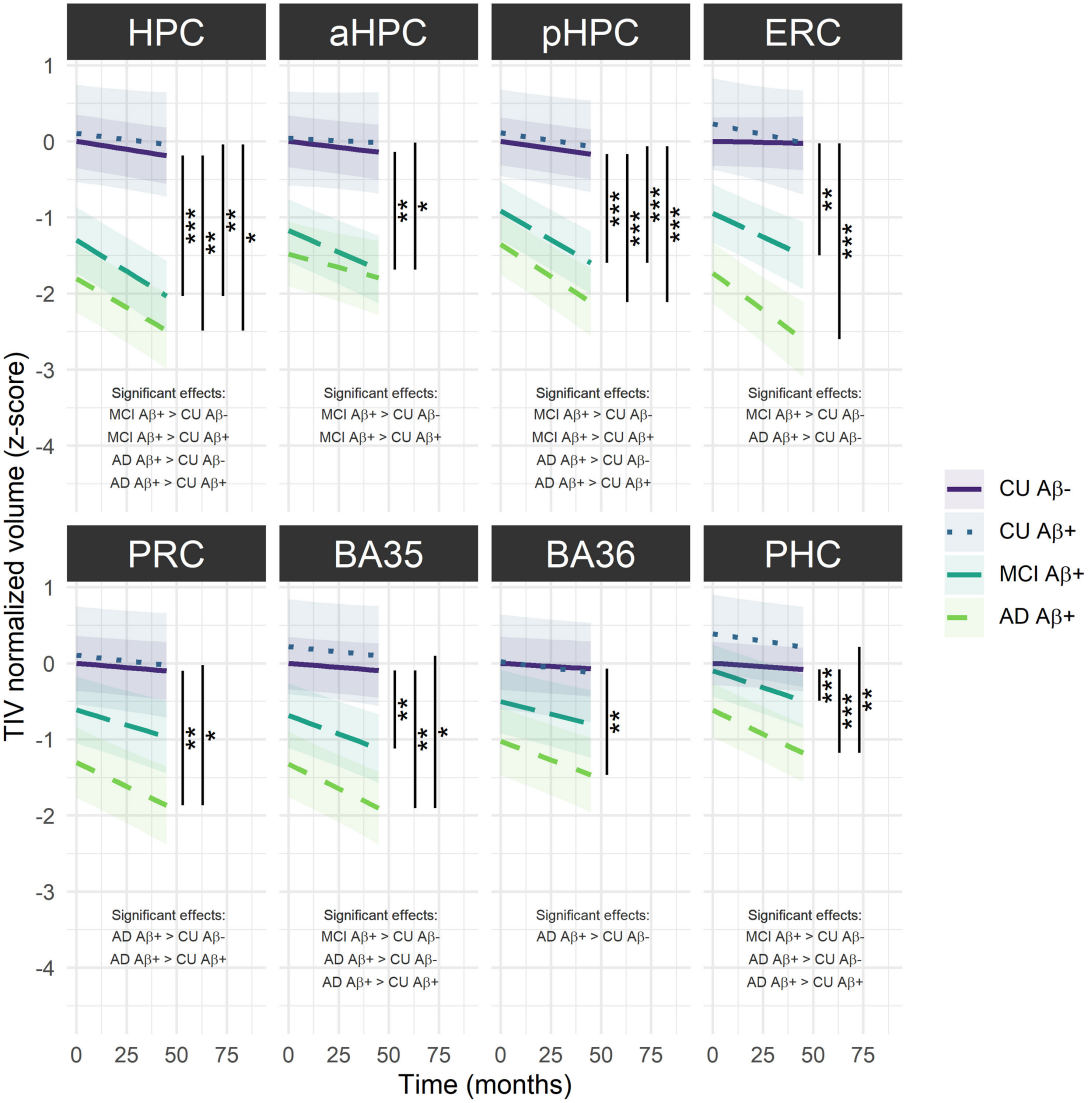
Group comparisons of MTL subregions volume decline over time across the Alzheimer's continuum. The regression line indicates model-derived estimates. The shaded area represents a 95% confidence interval. ***p < 0.001; **p < 0.01; *p < 0.05, as results of Tukey adjusted *post-hoc t*-tests. AD, Alzheimer's dementia; BA, Brodmann area; CU, cognitively unimpaired; ERC, entorhinal cortex; HPC, hippocampus (a, anterior; p, posterior); MCI, mild cognitive impairment; PRC, parahippocampal cortex; PRC, perirhinal cortex. The corresponding statistics are depicted in Supplementary Table 6.

For longitudinal analyses, the interaction *group* × *time* was significant for all the LMMs, suggesting that volume decline differs along the Alzheimer's continuum [HPC: *F*_(69.55)_ = 11.23, *p* < 0.001; aHPC: *F*_(85.82)_ = 4.46, *p* < 0.01; pHPC: *F*_(126.55)_ = 18.37, *p* < 0.001; ERC: *F*_(87.62)_ = 9.67, *p* < 0.001; PRC: *F*_(77.39)_ = 6.21, *p* < 0.001; BA35: *F*_(93.93)_ = 7.28, *p* < 0.001; BA36: *F*_(82.10)_ = 4.71, *p* < 0.01; PHC: *F*_(85.39)_ = 14.26, *p* < 0.001]. As illustrated in Figure 3, Aβ+ MCI patients showed significantly smaller HPC, aHPC, pHPC, ERC, BA35 and PHC volumes compared to Aβ− CU, and significantly smaller HPC, aHPC and pHPC volumes compared to Aβ+ CU. Aβ+ AD patients showed significantly smaller HPC, pHPC, ERC, PRC, BA35, BA36 and PHC volumes compared to Aβ− CU, and significantly smaller HPC, pHPC, PRC, BA35 and PHC volumes compared to Aβ+ CU. No significant differences between Aβ− and Aβ+ CU or between Aβ+ MCI and AD patients were observed (Supplementary Table 6).

### Volume Decline in Hippocampal Substructures Differs Between MCI-To-AD Converters and Non-converters

**Figure 4:**
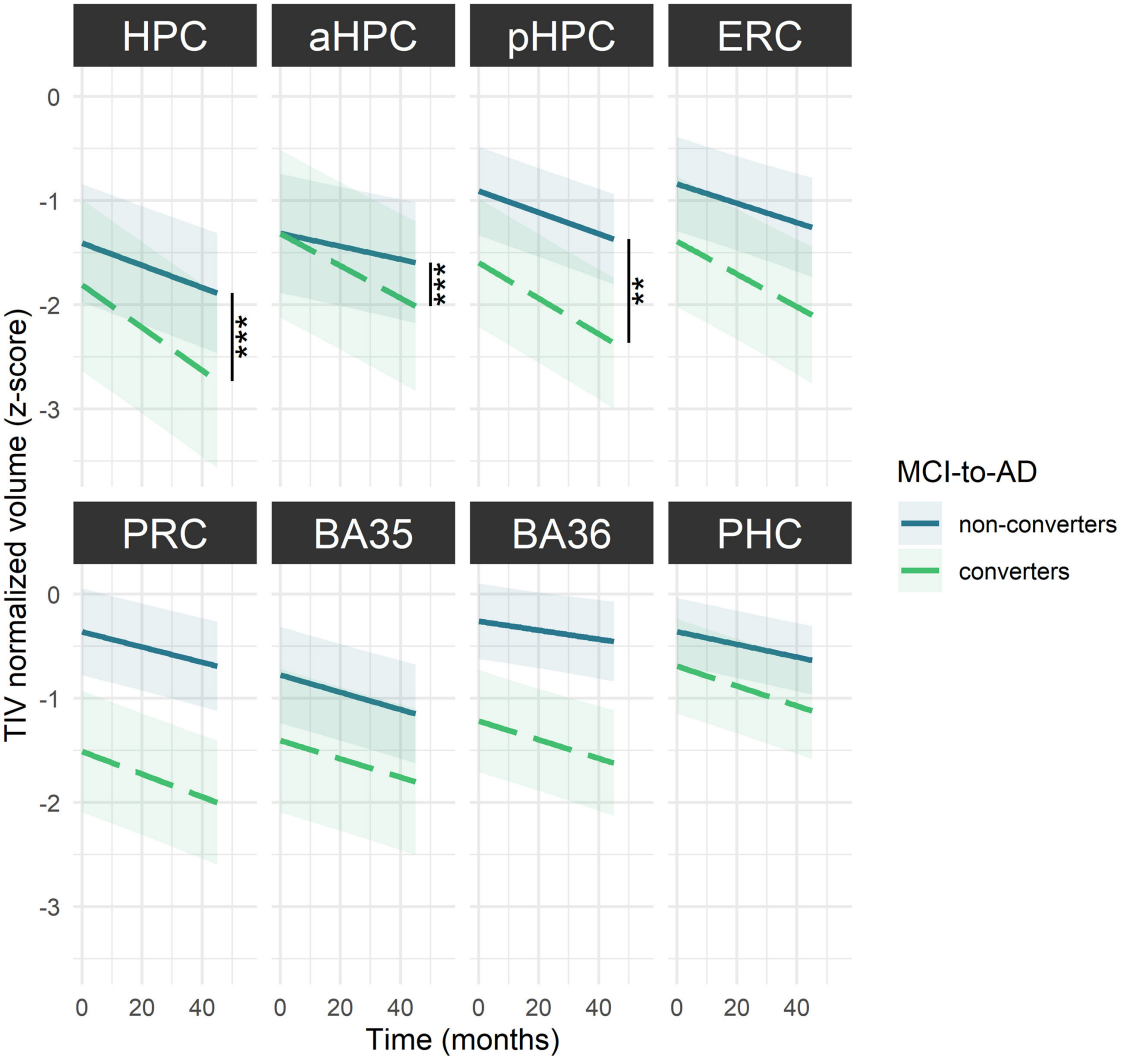
MTL subregions volume decline over time among MCI-to-AD converters and non-converters. The regression line indicates model-derived estimates. The shaded area represents a 95% confidence interval. ***p < 0.001; **p < 0.01, as results of F-tests after the Holm correction. BA, Brodmann area; ERC, entorhinal cortex; HPC, hippocampus (a, anterior; p, posterior); MCI, mild cognitive impairment; PHC, parahippocampal cortex; PRC, perirhinal cortex. The corresponding statistics are depicted in Supplementary Table 8.

Baseline volumes were not significantly different between MCI-to-AD converters and non-converters for any MTL subregion (Supplementary Table 7). However, converters showed significantly steeper volume decrease over time in hippocampal substructures compared to non-converters [HPC: *F*_(56.14)_ = 15.09, *p* < 0.001; aHPC: *F*_(58.21)_ = 12.44, *p* < 0.001 and pHPC: *F*_(56.13)_ = 9.28, *p* = 0.003) (Figure 4, Supplementary Table 8).

### MTL Subregions Are Specifically Associated With Episodic Memory Performances

Volumes in most MTL subregions were positively associated with episodic memory scores (HPC: β = 0.17, SE = 0.06, *p* = 0.006; pHPC: β = 0.21, SE = 0.08, *p* = 0.009; ERC: β = 0.22, SE = 0.07, *p* = 0.003; PRC: β = 0.20, SE = 0.07, *p* = 0.007; and BA35: β = 0.18, SE = 0.06, *p* = 0.008) but not with executive function or speed processing (Supplementary Table 9).

## Discussion

The present study aims to explore how physiological aging and AD affect the volume of MTL subregions. Our current contribution represents one of the most comprehensive studies on MTL volumetric data using a tailored methodology based on longitudinal data and a dedicated segmentation algorithm. The findings highlight differential effects of age over the adult lifespan and support that AD-related neurodegeneration is more severe in the late stages of the Alzheimer's continuum, reflecting cognitive impairment. Consistently, episodic memory deficit, a key feature of AD, is related to greater atrophy in most MTL subregions. Interestingly, subregions that undergo specific aging mechanisms, as evidence by non-linear age-related volume trajectories, although not different between Aβ− and Aβ+ CU older adults, are particularly affected from the prodromal stage of AD (i.e., Aβ+ MCI). This volume decline in the hippocampal substructures might also give information regarding the conversion to AD-dementia in patients with MCI.

### Differential Vulnerabilities of MTL Subregions in Aging

Our results emphasized that MTL subregions differ in their relationships with age over the adult lifespan, with both linear and non-linear age-related trajectories. Linear trajectories were observed for the first time for the PRC (as well as for BA 35 and 36) and PHC volumes, filling a knowledge gap regarding usually neglected MTL subregions. In addition, non-linear trajectories were observed for the HPC (and its anterior and posterior parts) and ERC volumes, strengthening the importance of not restricting analyses to linear models when investigating the effects of age (Chen et al., 2016). In line with previous findings, visual inspection of the trajectories indicates that the HPC and ERC volumes remained stable or expanded until middle age and then declined substantially (Ziegler et al., 2012; Fjell et al., 2013; Coupé et al., 2017; Amlien et al., 2018; Li et al., 2018; Nobis et al., 2019; Langnes et al., 2020; Nyberg et al., 2021). As entorhinal cortical-hippocampal circuits are important in episodic memory processing, this pattern might partly explain the similar trajectory of age-related episodic memory deficit (Rönnlund et al., 2005; Nyberg et al., 2012). Interestingly, the HPC and ERC are strongly involved in neuroplasticity-related mechanisms such as neurogenesis or spinal plasticity (Teter and Ashford, 2002; Neves et al., 2008; Fjell et al., 2014; Yun et al., 2018; Ronaghi et al., 2019). Because these processes are partly dependent on environmental factors, non-linear trajectories in the HPC and ERC volumes might reflect age-related changes in cellular morphology influenced by exogenous events experienced during a lifetime. When compared visually, the HPC and ERC volumes seem to decrease faster from midlife than those of the PRC and PHC, suggesting that they are more vulnerable to late aging; and this vulnerability might be the result of greater plasticity, in line with the proposal of McEwen (1994). Based on the approximation of the decline onset age, the ERC volume was found to decrease earlier than the HPC volume. One speculation behind this observation might be that ERC alterations trigger damage to the HPC, as these structures are strongly connected (Whitwell et al., 2007).

Interestingly, vulnerability to age also seems to vary along the HPC longitudinal axis. Indeed, the pHPC volume declined more severely than the aHPC, suggesting that aging preferentially affects the posterior part of the HPC. This anteroposterior specificity might explain why semantic memory is relatively spared in aging while episodic memory and spatial learning are more damaged (Brickman and Stern, 2009), as semantic memory is particularly associated with the aHPC while episodic memory and spatial navigation are preferentially associated with the pHPC (Ranganath and Ritchey, 2012). Our observations support previous results emphasizing less volume alteration in the aHPC compared with pHPC in typical aging (Kalpouzos et al., 2009), but is in contrast with the results showing comparable volume declines (Li et al., 2018) or greater aHPC vulnerability (Chen et al., 2010; Ta et al., 2012). Several methodological differences might explain these discrepancies. For example, the use of longitudinal vs. cross-sectional data might enhance the results reliability, especially since HPC volume varies greatly among individuals (Lupien et al., 2007). Age range, segmentation procedure, and curvilinear pattern might as well account for differences. However, such an anteroposterior gradient of vulnerability was not systematically found in studies comparable to ours (Langnes et al., 2020). Thus, differences between samples might also contribute to the discrepant results because age-related anatomical findings highly depend on inter-individual variance (Ta et al., 2012). From this perspective, CU participants over 60 years old included in our sample were highly educated (12.14 ± 2.85) compared with their peers born in the same period (~1,940), of whom only 20% had a high school diploma (Clerc et al., 2011), and probably belong to high socioeconomic backgrounds. Following the hypothesis of Baum et al. (1999) stating that stress might be the pathway linking socioeconomic status and health, it is possible that high socioeconomic status is associated with less exposure to stressful life events and might prevent individuals from a drastic aHPC volume decline, as the aHPC is particularly sensitive to stress (Vythilingam et al., 2005; Szeszko et al., 2006; Hawley and Leasure, 2012; Hawley et al., 2012; Decker et al., 2020). Further investigations of aHPC and pHPC volume trajectories across the lifespan are needed to draw strong conclusions, and the inclusion of a measure of stressful life events might help explore this possibility. Structural differentiation along the long axis of the HPC was also found in the first decades of the adult lifespan, as the aHPC volume still increased in early adulthood compared with the pHPC volume. This observation is in line with the mean diffusion results indicating that the pHPC microstructural development is completed during childhood whereas the aHPC still develops afterward (Langnes et al., 2020).

Overall, the differential vulnerabilities to age in MTL subregions do not seem to be influenced by sex, *APOE4*, or educational level. While investigating sex differences is not the main focus of this paper, we pointed out that two MTL subregions—pHPC and PHC—exhibited a larger adjusted volume in women than in men. This observation corroborates previous findings, highlighting a larger pHPC in women when adjusted for TIV (Persson et al., 2014; van Eijk et al., 2020). This anteroposterior specificity in sex differences might explain previous inconsistent findings regarding the HPC volume sex differences due to the hippocampus being considered as a whole.

### AD Particularly Damages MTL Subregions From the MCI Stage

In line with previous findings, MTL subregional atrophy differed along the Alzheimer's continuum, with later clinical stages being more affected (Wolk et al., 2017; Xie et al., 2019, 2020). Specifically, baseline volumes were significantly greater in CU older adults (either Aβ− or Aβ+) vs. Aβ+ AD patients for all MTL subregions, and vs. Aβ+ MCI patients only for HPC, aHPC, pHPC and ERC. Interestingly, adult lifespan findings describe these subregions as being affected late by the detrimental effects of aging, suggesting that the MTL subregions displaying a late age-related decline were impaired earlier in AD. In addition to significantly smaller baseline volumes, Aβ+ MCI and AD patients exhibited more severe volume decline over time for most of the MTL subregions compared to Aβ− CU older adults, illustrating the sensitivity of longitudinal analyses in tracking the progression of the disease. Given these findings, MTL subregional atrophy appears as a valid biomarker of the symptomatic stages of the Alzheimer's continuum and seems more widespread at the dementia stage, corroborating studies that have already proven the considerable alteration of the MTL in patients with MCI and AD (Juottonen et al., 1998; Du et al., 2001; Pennanen et al., 2004; Dickerson et al., 2009; Stoub et al., 2010; La Joie et al., 2012). The ERC and HPC (especially the pHPC) were identified as the most damaged subregions from the MCI stage, suggesting that AD preferentially targets long-development MTL subregions, as volume of these subregions keeps increasing until middle age. These subregions might be more vulnerable to the detrimental effects of late aging because of their high plasticity, and this age vulnerability might make them more susceptible to additional pathological AD-related changes, following the hypothesis described in Fjell et al. (2014). Thus, environmental factors that influence volume loss in aging could likely predispose to AD-related alterations. Interestingly, the MTL subregions most prone to atrophy in AD were also the most affected by tau pathology regarding tangles topography (Braak et al., 2006). These observations corroborate the relationship between atrophy and neurofibrillary tangles outlined in previous studies (Whitwell et al., 2008; Harrison et al., 2019; de Flores et al., 2020).

Although our results advocate the benefits of studying MTL subregions to differentiate patients of the Alzheimer's continuum from CU, none of these measures detect early pathological changes defined by the presence of Aβ deposition. Previous cross-sectional findings are inconsistent regarding the potential ability of MTL subregional volumes to differentiate between Aβ− and Aβ+ CU older adults. Some studies report no differences (Xie et al., 2019) while others found smaller BA 35 in Aβ+ CU (Wolk et al., 2017; Xie et al., 2020). Because these results were derived from cortical thickness measurements rather than volumetric measurements, we suppose that cortical thickness may be more sensitive in tracking early signs of AD, especially since extra-hippocampal volume measures are biased by the depth of the collateral sulcus which varies among individuals (Feczko et al., 2009; Schwarz et al., 2016; Berron et al., 2017). Still, the differences were small and did not survive the multiple testing correction. Interestingly, the longitudinal evaluation of MTL subregional atrophy seemed to enhance the clinical utility of these biomarkers. Xie et al. (2020) revealed more robust significant differences between Aβ− and Aβ+ CU older adults using longitudinal data, and these differences were extended to most MTL subregions, yet, we failed to replicate their findings in our analyses. As Xie et al. divided Aβ+ CU participants into tau+ and tau- subgroups and found that only the Aβ+ tau+ subgroup was significantly different from Aβ− CU older adults, we can speculate that most of the participants in our sample did not have significant tau pathology, so no MTL subregional atrophy could be detected. Unfortunately, no tau data was available in our study, so further studies including such measures will be needed to validate this assumption.

Neither the baseline nor longitudinal measures of MTL subregional atrophy dissociated Aβ+ MCI and AD patients, except for the ERC at baseline. This observation corroborates previous findings that found significantly reduced ERC volume in MCI compared with AD patients (Zhou et al., 2016 for review), but do not replicate other previous findings highlighting HPC volume difference between MCI and AD (Lu et al., 2019; Zhao et al., 2019).

Overall, MTL subregional atrophy appears to reflect AD-related cognitive impairments, as it distinguishes patients from CU older adults, but may not be an ideal biomarker of early AD-related pathological changes defined by the presence of Aβ deposits.

### Hippocampal Substructures Are Sensitive to Alzheimer's Dementia Conversion in MCI Patients

From a prognostic perspective, the HPC, aHPC, and pHPC volume declines were steeper in MCI-to-AD converters than in non-converters, suggesting that hippocampal substructures atrophy may provide information on the clinical outcomes of MCI patients. These observations corroborate previous findings also highlighting greater hippocampal atrophy in MCI patients who developed AD-dementia compared with those who did not (Pennanen et al., 2004; Apostolova et al., 2006; Devanand et al., 2007; Henneman et al., 2009; Maruszak and Thuret, 2014 for review; Brueggen et al., 2015). The sensitivity of hippocampal substructures in dissociating converters from non-converters was thought to reflect the important alteration of the HPC in AD. Because group differences were found only by comparing the volume declines over time and not the baseline volumes, a longitudinal assessment of atrophy appears to be more sensitive for tracking AD progression than single-time assessment, especially as cross-sectional measures may suffer from cohort effects. In addition, a standardized hippocampal atrophy score based on automatic segmentation procedures, with norms as for neuropsychological testing, might provide a more objective measurement than the subjective appreciation of MTL atrophy used in previous studies (Scheltens et al., 1995, 1997; Korf et al., 2004; Lehmann et al., 2013). In contrast to some studies that have illustrated the ability of ERC volume to predict conversion to AD-dementia in MCI patients, no significant dissociation between converters and non-converters was found for this subregion in our analyses (de Toledo-Morrell et al., 2004; Zhou et al., 2016 for reviews). Still, the ERC was the subregion displaying the greatest groups difference among non-significant models. To the best of our knowledge, the PRC (and its subcomponents BA 35 and 36) and PHC volume differences were investigated for the first time between MCI-to-AD converters and non-converters, and results suggest that none of these subregions discriminate converters from non-converters.

These results might sound promising as they consider a potential prognostic biomarker for AD conversion, but it must be remembered that this is a group comparison and therefore does not directly evaluate the predictive value of these measures. Further studies entirely dedicated to the predictive ability of MTL subregions volumetry to anticipate AD-dementia conversion in MCI patients might help establish a better clinical routine.

### The Volume of Most MTL Subregions Reflects Episodic Memory Performances

According to our hypotheses, we outlined robust relationships between most of MTL subregions volumes and episodic memory performances in Aβ+ MCI and AD patients, suggesting that pronounced MTL subregional atrophy worsens episodic memory. Relative to the cognitive functions investigated, the association between episodic memory and MTL subregions volume was domain-specific, but very few cognitive areas were assessed, which limits our interpretation. Further studies including a more comprehensive assessment of cognitive functioning might complement our results and provide more substantiated information. In particular, indexes of semantic and spatial memory may be relevant, as some MTL subregions are specifically involved in these processes.

Our results suggested that the pHPC, ERC, and PRC volumes were the most strongly associated with episodic memory performances. These MTL subregions were also the most damaged in the late clinical stages of the Alzheimer's continuum, and so substantiate the view of episodic memory deficit as a core feature of AD. Thus, the strong involvement of HPC and ERC in episodic memory function might explain why episodic memory may be enriched by our environment as we suggested that environmental factors could impact their development. Rather, the aHPC, BA36, and PHC volumes were not significantly associated with episodic memory function. The lack of association between PHC volumes and episodic memory performances was quite expected as PHC is more involved in visuospatial processing (Ranganath and Ritchey, 2012; Aminoff et al., 2013; Bohbot et al., 2015; Baumann and Mattingley, 2021). However, the role played by the PHC in episodic memory should not be negated as it is known that PHC supports the representations of the situational context associated with items (Ranganath and Ritchey, 2012; Aminoff et al., 2013). Indeed, our scores might not be sensitive enough to capture the associative processing aspect of episodic memory since tests often fail to capture all reality subtleties, such as contextual details. Thus, new tests assessing episodic memory more realistically have been designed and might be explored to better reflect the complexity of episodic memory (Curot, 2018; Smith, 2019). Interestingly, the aHPC volume was less related to episodic memory performances compared with the pHPC volume. This disparity within hippocampal substructures may reflect the differential role of the two MTL networks, as aHPC belongs to an anterior-temporal network which is known to be more involved in semantic memory compared with pHPC which is included in a posterior-medial network that is preferentially involved in episodic memory (Ranganath and Ritchey, 2012).

### Limitations

The present study has some limitations. First, it should be mentioned that the age of our lifespan sample, ranging from 19 and 85, limits the interpretability of age-related changes in the early lifespan. Specifically, the first inflection in the HPC volume trajectory should be considered with caution, and additional analyses including children and adolescents are needed to refine our observations. Also, our sample size can appear relatively small and might prevent us from showing subtle effects, especially early structural changes in preclinical AD, as only the data from 14 Aβ+ CU older adults were available. However, all data used in this article were acquired using the same MRI scanner and this sample size allowed us to rigorously check the scans quality and manually edit the segmentations, thus, greatly improving the accuracy of our data. In addition, we manually corrected about 30% of the segmentations, which may suggest difficulties for MTL volumetry clinical use. However, it is important to note that most of these errors were found in CU young adults, certainly reflecting the inclusion lack of such an age group in the atlas used. Thus, modifying the atlas to better match a specific targeted clinical population would likely optimize the software segmentations.

## Conclusion

Through this longitudinal study, we gained a better understanding of aging and AD-related mechanisms regarding the volume of MTL subregions. We emphasized the benefits of studying these biomarkers to distinguish age-related changes from AD. Interestingly, the MTL subregions were differently vulnerable to the detrimental effects of aging, and the subregions displaying a late-onset decline, while not affected by the presence of Aβ deposits, were particularly damaged in AD from the MCI stage, as well as closely related to episodic memory performances. In particular, the volume decline in the hippocampal substructures might predict the conversion from MCI to AD-dementia. Overall, we hope that these findings will provide new insights into MTL alterations, which are crucial for the definition of AD-specific biomarkers.

## Data Availability Statement

The raw data supporting the conclusions of this article will be made available by the authors, without undue reservation.

## Ethics Statement

The studies involving human participants were reviewed and approved by Comité de Protection des Personnes Nord-Ouest III. The patients/participants provided their written informed consent to participate in this study.

## Author Contributions

GC and VdLS were the principal investigators of the IMAP+ research protocol. CP, FF, and VO contributed to the data acquisition. RdF and LC conceived the study. LC executed the study and was responsible for data analysis and data interpretation. RdF and EK contributed to the data analysis and data interpretation. LC drafted the manuscript in close collaboration with RdF. GC, EK, VdLS, CP, FF, and VO provided feedback on the manuscript. All authors have read and approved the manuscript, and so contributed substantially to it.

## Funding

This work was supported by the Programme Hospitalier de Recherche Clinique (PHRCN 2011-A01493-38 and PHRCN 2012 12-006-0347), the Agence Nationale de la Recherche (ANR LONGVIE 2007), Fondation Plan Alzheimer (Alzheimer Plan 2008–2012), Association France Alzheimer et maladies apparentées AAP 2013, the Région Basse Normandie, and the Institut National de la Santé et de la Recherche Médicale (INSERM).

## Conflict of Interest

The authors declare that the research was conducted in the absence of any commercial or financial relationships that could be construed as a potential conflict of interest.

## Publisher's Note

All claims expressed in this article are solely those of the authors and do not necessarily represent those of their affiliated organizations, or those of the publisher, the editors and the reviewers. Any product that may be evaluated in this article, or claim that may be made by its manufacturer, is not guaranteed or endorsed by the publisher.

## Abbreviations

AD: Alzheimer's disease
BA: Brodmann area
CU: cognitively unimpaired
ERC: entorhinal cortex
HPC: hippocampus (a, anterior; p, posterior)
LMM: linear mixed model
MCI: mild cognitive impairment
MRI: magnetic resonance imaging
PHC: parahippocampal cortex
PRC: perirhinal cortex
TIV: total intracranial volume.
