# Supplementary material for: Medial Temporal Lobe Subregional Atrophy in Aging and Alzheimer's Disease: A Longitudinal Study
Source: Front Aging Neurosci. 2021 Oct 15;13:750154. doi: 10.3389/fnagi.2021.750154 (PMC8554299; doi:10.3389/fnagi.2021.750154)
Supplement: Supplementary file 1 [file Data_Sheet_1.PDF]

Supplementary Material

1 Supplementary Method

1.1 Model selection for age-related trajectories

Age-term was modelled with a polynomial of degree *1* to *n+1* where *n* is the best-fit degree. Different model types were considered to estimate the final model for each MTL-subregion. The candidate models were tested from the simplest to the most complex. A model type was kept as candidate when likelihood ratio test (polynomial of degree *n* versus polynomial of degree *n-1*) and all age term coefficients were significant using t-statistic (P<0.05). The $\Delta_{AICc/BIC} > 10$ (as the difference between *n* and *n-1* models for AICc/BIC values) was considered as a robust sign that the model with lower AICc/BIC should be preferred (Burnham and Anderson, 2004). For differences whose interpretation was less straightforward, the ratio likelihood test was preferred to test whether the more complex model was significantly better at capturing the data than the simpler model. We noted that the selection of a cubic polynomial for the hippocampus is less evident and first inflection point should be interpreted with caution as AICc and BIC tend towards divergent conclusions (probably because BIC penalizes model complexity more heavily) and p-value of the likelihood ratio test is close to significance threshold of 0.05 (increasing the probability of type I error). Since youngest participants included in the dataset were 19 years old, it was difficult to robustly infer a change around 21 years old. Thus, further analysis in children and teenagers would help to better understand age-related trajectory of hippocampal volume during this early adulthood.

2 Supplementary Figures and Tables

2.1 Supplementary Tables

Supplementary Table 1. Model comparison for the age effect on MTL subregions volume. Model in bold is the selected model. *AICc, Akaike information criterion corrected; BA, Brodmann area; BIC, Bayesian information criterion; ERC, Entorhinal cortex; HPC, Hippocampus (a, anterior; p, posterior); MTL, medial temporal lobe; LRT, likelihood ratio test; PHC, Parahippocampal cortex; PRC, Perirhinal cortex.*

	model	t-test of each coefficient ($\hat{c}$) for the age term *(p-value)*				LRT *(p-value)*	AICc	BIC
		$\hat{c}_1$	$\hat{c}_2$	$\hat{c}_3$	$\hat{c}_4$			
HPC	m_1	**0.000**	-	-	-	-	418.95	442.88
	m_2	**0.001**	**0.000**	-	-	**0.000**	397.76	425.64
	**m_3**	**0.003**	**0.000**	**0.037**	-	**0.036**	395.46	427.29
	m_4	**0.004**	**0.000**	**0.036**	0.575	0.575	397.24	433.00
aHPC	m_1	0.125	-	-	-	-	400.41	424.38
	**m_2**	0.227	**0.000**	-	-	**0.000**	388.85	416.77
	m_3	0.343	**0.000**	0.356	-	0.356	390.07	421.94
pHPC	m_1	**0.000**	-	-	-	-	418.11	442.04
	m_2	**0.000**	**0.000**	-	-	**0.000**	403.16	431.05
	**m_3**	**0.000**	**0.000**	**0.009**	-	**0.009**	398.45	430.28
	m_4	**0.000**	**0.001**	**0.009**	0.129	0.132	398.27	434.03
ERC	m_1	**0.000**	-	-	-	-	598.45	622.36
	**m_2**	**0.000**	**0.000**	-	-	**0.000**	583.53	611.38
	m_3	**0.000**	**0.000**	0.715	-	0.715	585.48	617.27
PRC	**m_1**	**0.000**	-	-	-	-	269.19	292.63
	m_2	**0.000**	0.244	-	-	297.216	269.92	297.22
BA35	**m_1**	**0.000**	-	-	-	-	504.53	528.49
	m_2	**0.000**	0.087	-	-	0.087	503.67	531.59
BA36	**m_1**	**0.000**	-	-	-	-	208.67	232.12
	m_2	**0.000**	0.767	-	-	0.768	210.66	237.98
PHC	**m_1**	**0.000**	-	-	-	-	238.40	262.32
	m_2	**0.000**	0.262	-	-	-	239.21	267.08

Supplementary Table 2. Effect of sex on MTL subregions volume in cognitively unimpaired adults. Statistic summary of the main effect *sex* and the interaction effect *age* x *sex* from LMMs, as the result of model testing with F test (sample size [n], F-values, approximate denominator degrees of freedom [ddf] and p-values are reported). Bolded p-values survived the Holm correction (*P*<.05). *BA, Brodmann area; ERC, Entorhinal cortex; HPC, Hippocampus (a, anterior; p, posterior); MTL, medial temporal lobe; PHC, Parahippocampal cortex; PRC, Perirhinal cortex.*

	Effect	ddf	F	*P*	n
HPC	sex	197,78	6,33	0,0127	208
	age x sex	307,87	0,94	0,4214	
aHPC	sex	201,05	2,06	0,1525	209
	age x sex	335,01	1,45	0,2351	
pHPC	sex	202,88	7,94	**0,0053**	208
	age x sex	311,41	1,03	0,3809	
ERC	sex	199,16	1,67	0,1981	207
	age x sex	302,90	0,01	0,9932	
PRC	sex	186,22	0,00	0,9803	190
	age x sex	305,71	0,29	0,5932	
BA35	sex	206,16	5,92	0,0158	209
	age x sex	278,90	2,07	0,1518	
BA36	sex	187,99	0,33	0,5645	191
	age x sex	328,49	1,18	0,2774	
PHC	sex	204,66	23,38	**0,0000**	207
	age x sex	358,06	0,01	0,9423	

Supplementary Table 3. Effect of education on MTL subregions volume in cognitively unimpaired adults. Statistic summary of the main effect *education* and the interaction effect *age* x *education* from LMMs, as the result of model testing with F test (sample size [n], F-values, approximate denominator degrees of freedom [ddf] and p-values are reported). None of all considered MTL-subregions showed neither significantly different age-related trajectories nor significantly different volumes by the education level (P<.05). *BA, Brodmann area; ERC, Entorhinal cortex; HPC, Hippocampus (a, anterior; p, posterior); MTL, medial temporal lobe; PHC, Parahippocampal cortex; PRC, Perirhinal cortex.*

	Effect	ddf	F	P	n
HPC	education	337,85	1,02	0,3845	208
	education x age	300,04	1,34	0,2478	
aHPC	education	358,93	0,52	0,5937	209
	education x age	308,44	0,64	0,4250	
pHPC	education	333,87	0,81	0,4867	208
	education x age	303,81	1,19	0,2766	
ERC	education	361,14	0,61	0,5427	207
	education x age	256,70	2,18	0,1412	
PRC	education	369,30	1,01	0,3157	190
	education x age	291,46	1,07	0,3027	
BA35	education	367,34	0,33	0,5679	209
	education x age	283,67	0,00	0,9470	
BA36	education	376,70	0,63	0,4294	191
	education x age	309,18	1,06	0,3035	
PHC	education	407,99	0,37	0,5433	207
	education x age	347,63	0,35	0,5541	

Supplementary Table 4. Effect of *APOE4* on MTL subregions volume in cognitively unimpaired adults. Statistic summary of the main effect *apoe* and the interaction effect *age* x *apoe* from LMMs, as the result of model testing with F test (sample size [n], F-values, approximate denominator degrees of freedom [ddf] and p-values are reported). None of all considered MTL-subregions showed neither significantly different age-related trajectories nor significantly different volumes by *APOE* genotype (P<.05). *BA, Brodmann area; ERC, Entorhinal cortex; HPC, Hippocampus (a, anterior; p, posterior); MTL, medial temporal lobe; PHC, Parahippocampal cortex; PRC, Perirhinal cortex.*

	Effect	ddf	F	*P*	n
HPC	apoe	313,70	1,23	0,2979	202
	apoe x age	198,16	0,09	0,7652	
aHPC	apoe	333,44	1,22	0,2964	203
	apoe x age	199,88	0,76	0,3838	
pHPC	apoe	312,75	0,85	0,4675	202
	apoe x age	199,10	0,20	0,6559	
ERC	apoe	296,85	0,89	0,4131	201
	apoe x age	195,00	0,52	0,4711	
PRC	apoe	284,62	0,88	0,3498	185
	apoe x age	182,47	0,09	0,7658	
BA35	apoe	270,59	0,02	0,8958	203
	apoe x age	200,54	2,94	0,0880	
BA36	apoe	303,74	1,11	0,2920	185
	apoe x age	182,58	1,22	0,2708	
PHC	apoe	352,95	0,03	0,8670	201
	apoe x age	199,09	0,26	0,6130	

Supplementary Table 5. MTL subregions baseline volume across the Alzheimer's continuum. Left: Statistic summary of the *group* main effect from multiple regression, as the result of model testing with F test (sample size [n], F-values, degrees of freedom [df], p-values and adjusted R^2 are reported). Bolded p-values survived the Holm correction (P<.05). Middle: Estimated marginal means of baseline volume for each group (estimates and 95% confidence interval [CI] are reported). Right: Statistic Summary of *post-hoc* pairwise comparisons (estimates, standard errors [SE], t-ratios and Tukey adjusted p-values are reported). *AD, Alzheimer's dementia; CU, cognitively unimpaired; BA, Brodmann area; ERC, Entorhinal cortex; HPC, Hippocampus (a, anterior; p, posterior); MCI, mild cognitive impairment*; *MTL, medial temporal lobe; PHC, Parahippocampal cortex; PRC, Perirhinal cortex.*

	Main effect: *group*					Baseline volume				Pairwise comparisons				
	df	F	P	R^2	n	group	emmean	95% CI		contrast	estimate	SE	t	P
HPC	3	19,32	**3,1E-10**	0,32	123	CU Aβ-	-0,062	-0,419	0,295	(CU Aβ-) - (CU Aβ+)	-0,113	0,375	-0,30	0,9906
						CU Aβ+	0,050	-0,595	0,696	(CU Aβ-) - (MCI Aβ+)	1,293	0,290	4,46	**0,0001**
						MCI Aβ+	-1,355	-1,792	-0,918	(CU Aβ-) - (AD Aβ+)	1,797	0,287	6,27	**0,0000**
						AD Aβ+	-1,859	-2,302	-1,417	(CU Aβ+) - (MCI Aβ+)	1,406	0,388	3,62	**0,0024**
										(CU Aβ+) - (AD Aβ+)	1,910	0,401	4,76	**0,0000**
										(MCI Aβ+) - (AD Aβ+)	0,504	0,320	1,58	0,3964
aHPC	3	14,25	**5,6E-08**	0,25	124	CU Aβ-	-0,043	-0,388	0,301	(CU Aβ-) - (CU Aβ+)	-0,031	0,361	-0,09	0,9998
						CU Aβ+	-0,012	-0,634	0,610	(CU Aβ-) - (MCI Aβ+)	1,176	0,276	4,26	**0,0002**
						MCI Aβ+	-1,220	-1,632	-0,807	(CU Aβ-) - (AD Aβ+)	1,489	0,276	5,39	**0,0000**
						AD Aβ+	-1,532	-1,958	-1,106	(CU Aβ+) - (MCI Aβ+)	1,208	0,372	3,25	**0,0081**
										(CU Aβ+) - (AD Aβ+)	1,520	0,386	3,93	**0,0008**
										(MCI Aβ+) - (AD Aβ+)	0,312	0,304	1,03	0,7351
pHPC	3	14,04	**7,2E-08**	0,27	123	CU Aβ-	-0,056	-0,374	0,262	(CU Aβ-) - (CU Aβ+)	-0,138	0,334	-0,41	0,9760
						CU Aβ+	0,082	-0,492	0,656	(CU Aβ-) - (MCI Aβ+)	0,897	0,258	3,48	**0,0040**
						MCI Aβ+	-0,953	-1,341	-0,564	(CU Aβ-) - (AD Aβ+)	1,334	0,255	5,23	**0,0000**
						AD Aβ+	-1,390	-1,783	-0,996	(CU Aβ+) - (MCI Aβ+)	1,035	0,345	3,00	**0,0172**
										(CU Aβ+) - (AD Aβ+)	1,472	0,357	4,13	**0,0004**
										(MCI Aβ+) - (AD Aβ+)	0,437	0,284	1,54	0,4188
ERC	3	17,36	**2,2E-09**	0,31	123	CU Aβ-	-0,031	-0,356	0,293	(CU Aβ-) - (CU Aβ+)	-0,234	0,351	-0,67	0,9098
						CU Aβ+	0,203	-0,406	0,812	(CU Aβ-) - (MCI Aβ+)	0,948	0,260	3,64	**0,0023**
						MCI Aβ+	-0,979	-1,368	-0,590	(CU Aβ-) - (AD Aβ+)	1,726	0,260	6,63	**0,0000**
						AD Aβ+	-1,757	-2,158	-1,356	(CU Aβ+) - (MCI Aβ+)	1,182	0,359	3,29	**0,0071**
										(CU Aβ+) - (AD Aβ+)	1,960	0,374	5,24	**0,0000**
										(MCI Aβ+) - (AD Aβ+)	0,778	0,287	2,71	**0,0382**
PRC	3	7,09	**2,2E-04**	0,17	110	CU Aβ-	-0,031	-0,396	0,334	(CU Aβ-) - (CU Aβ+)	-0,097	0,378	-0,26	0,9941

						CU Aβ+	0,066	-0,584	0,716	(CU Aβ-) - (MCI Aβ+)	0,636	0,294	2,16	0,1406
						MCI Aβ+	-0,667	-1,109	-0,225	(CU Aβ-) - (AD Aβ+)	1,316	0,297	4,44	**0,0001**
						AD Aβ+	-1,347	-1,810	-0,884	(CU Aβ+) - (MCI Aβ+)	0,732	0,391	1,87	0,2468
										(CU Aβ+) - (AD Aβ+)	1,412	0,406	3,48	**0,0041**
										(MCI Aβ+) - (AD Aβ+)	0,680	0,327	2,08	0,1663
BA35	3	9,16	**1,7E-05**	0,16	122	CU Aβ-	-0,009	-0,358	0,340	(CU Aβ-) - (CU Aβ+)	-0,189	0,367	-0,52	0,9551
						CU Aβ+	0,180	-0,451	0,812	(CU Aβ-) - (MCI Aβ+)	0,706	0,284	2,49	0,0676
						MCI Aβ+	-0,715	-1,144	-0,285	(CU Aβ-) - (AD Aβ+)	1,335	0,280	4,77	**0,0000**
						AD Aβ+	-1,344	-1,775	-0,913	(CU Aβ+) - (MCI Aβ+)	0,895	0,380	2,36	0,0918
										(CU Aβ+) - (AD Aβ+)	1,524	0,392	3,89	**0,0010**
										(MCI Aβ+) - (AD Aβ+)	0,629	0,312	2,02	0,1869
BA36	3	4,56	**4,8E-03**	0,13	111	CU Aβ-	-0,036	-0,388	0,315	(CU Aβ-) - (CU Aβ+)	-0,024	0,365	-0,07	0,9999
						CU Aβ+	-0,013	-0,640	0,615	(CU Aβ-) - (MCI Aβ+)	0,520	0,281	1,85	0,2566
						MCI Aβ+	-0,556	-0,976	-0,136	(CU Aβ-) - (AD Aβ+)	1,035	0,286	3,61	**0,0026**
						AD Aβ+	-1,071	-1,519	-0,623	(CU Aβ+) - (MCI Aβ+)	0,543	0,376	1,44	0,4746
										(CU Aβ+) - (AD Aβ+)	1,059	0,392	2,70	**0,0400**
										(MCI Aβ+) - (AD Aβ+)	0,515	0,314	1,64	0,3592
PHC	3	3,96	**1,0E-02**	0,14	124	CU Aβ-	-0,088	-0,376	0,200	(CU Aβ-) - (CU Aβ+)	-0,399	0,303	-1,32	0,5530
						CU Aβ+	0,311	-0,210	0,832	(CU Aβ-) - (MCI Aβ+)	0,111	0,231	0,48	0,9631
						MCI Aβ+	-0,199	-0,545	0,146	(CU Aβ-) - (AD Aβ+)	0,615	0,231	2,66	**0,0436**
						AD Aβ+	-0,703	-1,059	-0,346	(CU Aβ+) - (MCI Aβ+)	0,510	0,311	1,64	0,3603
										(CU Aβ+) - (AD Aβ+)	1,014	0,323	3,13	**0,0115**
										(MCI Aβ+) - (AD Aβ+)	0,504	0,255	1,98	0,2029

Supplementary Table 6. MTL-subregions volume decline across the Alzheimer's continuum. Left: Statistic summary of the *group* x *time* interaction effect from LMMs, as the result of model testing with F test (sample size [n], F-values, approximate denominator degrees of freedom [ddf], p-values and marginal R^2 [i.e., approximation of the variance explained by the fixed effects] are reported). Bolded p-values survived the Holm correction ($P<.05$). Middle: Estimated marginal trends of volume decline for each group (estimates and 95% confidence interval [CI] are reported). Right: Statistic Summary of *post-hoc* pairwise comparisons (estimates, standard errors [SE], t-ratios and Tukey adjusted p-values are reported). *AD, Alzheimer's dementia; CU, cognitively unimpaired; BA, Brodmann area; ERC, Entorhinal cortex; HPC, Hippocampus (a, anterior; p, posterior); MCI, mild cognitive impairment*; *MTL, medial temporal lobe; PHC, Parahippocampal cortex; PRC, Perirhinal cortex.*

	Interaction effect: *group* x *time*					Decline				Pairwise comparisons				
	ddf	F	*P*	R^2m	n	group	emtrend	95% CI		contrast	estimate	SE	t	*P*
HPC	69,55	11,23	**4,3x10^{-6}**	0,37	123	CU Aβ–	-0,0042	-0,0069	-0,0015	(CU Aβ–) - (CU Aβ+)	-0,0010	0,0033	-0,30	0,9910
						CU Aβ+	-0,0032	-0,0092	0,0027	(CU Aβ–) - (MCI Aβ+)	0,0122	0,0025	4,84	**0,0000**
						MCI Aβ+	-0,0164	-0,0206	-0,0122	(CU Aβ–) - (AD Aβ+)	0,0110	0,0031	3,59	**0,0027**
						AD Aβ+	-0,0152	-0,0207	-0,0098	(CU Aβ+) - (MCI Aβ+)	0,0132	0,0037	3,60	**0,0031**
										(CU Aβ+) - (AD Aβ+)	0,0120	0,0041	2,95	**0,0197**
										(MCI Aβ+) - (AD Aβ+)	-0,0012	0,0035	-0,34	0,9865
aHPC	85,82	4,46	**5,8x10^{-3}**	0,28	124	CU Aβ–	-0,0032	-0,0059	-0,0004	(CU Aβ–) - (CU Aβ+)	-0,0019	0,0034	-0,57	0,9403
						CU Aβ+	-0,0012	-0,0073	0,0049	(CU Aβ–) - (MCI Aβ+)	0,0082	0,0025	3,28	**0,0088**
						MCI Aβ+	-0,0114	-0,0156	-0,0072	(CU Aβ–) - (AD Aβ+)	0,0038	0,0032	1,18	0,6426
						AD Aβ+	-0,0070	-0,0127	-0,0012	(CU Aβ+) - (MCI Aβ+)	0,0102	0,0037	2,74	**0,0379**
										(CU Aβ+) - (AD Aβ+)	0,0057	0,0042	1,35	0,5316
										(MCI Aβ+) - (AD Aβ+)	-0,0044	0,0036	-1,24	0,6036
pHPC	126,55	18,37	**5,9x10^{-10}**	0,32	123	CU Aβ–	-0,0038	-0,0059	-0,0017	(CU Aβ–) - (CU Aβ+)	0,0001	0,0025	0,04	1,0000
						CU Aβ+	-0,0039	-0,0085	0,0007	(CU Aβ–) - (MCI Aβ+)	0,0114	0,0019	5,87	**0,0000**
						MCI Aβ+	-0,0152	-0,0185	-0,0119	(CU Aβ–) - (AD Aβ+)	0,0131	0,0025	5,17	**0,0000**
						AD Aβ+	-0,0169	-0,0215	-0,0123	(CU Aβ+) - (MCI Aβ+)	0,0113	0,0028	3,99	**0,0009**
										(CU Aβ+) - (AD Aβ+)	0,0130	0,0033	3,98	**0,0007**
										(MCI Aβ+) - (AD Aβ+)	0,0017	0,0028	0,60	0,9329
ERC	87,62	9,67	**1,4x10^{-5}**	0,36	123	CU Aβ–	-0,0006	-0,0042	0,0030	(CU Aβ–) - (CU Aβ+)	0,0049	0,0044	1,11	0,6864
						CU Aβ+	-0,0054	-0,0134	0,0025	(CU Aβ–) - (MCI Aβ+)	0,0117	0,0032	3,63	**0,0029**
						MCI Aβ+	-0,0123	-0,0176	-0,0070	(CU Aβ–) - (AD Aβ+)	0,0188	0,0040	4,71	**0,0000**
						AD Aβ+	-0,0193	-0,0264	-0,0123	(CU Aβ+) - (MCI Aβ+)	0,0068	0,0048	1,42	0,4923
										(CU Aβ+) - (AD Aβ+)	0,0139	0,0054	2,59	0,0521
										(MCI Aβ+) - (AD Aβ+)	0,0071	0,0044	1,59	0,3904

PRC	77,39	6,21	**7,7x10^{-4}**	0,21	110	CU Aβ–	-0,0023	-0,0046	0,0001	(CU Aβ–) - (CU Aβ+)	0,0007	0,0027	0,24	0,9948
						CU Aβ+	-0,0029	-0,0077	0,0019	(CU Aβ–) - (MCI Aβ+)	0,0057	0,0023	2,51	0,0668
						MCI Aβ+	-0,0079	-0,0118	-0,0041	(CU Aβ–) - (AD Aβ+)	0,0103	0,0027	3,85	**0,0011**
						AD Aβ+	-0,0126	-0,0174	-0,0078	(CU Aβ+) - (MCI Aβ+)	0,0050	0,0031	1,61	0,3770
										(CU Aβ+) - (AD Aβ+)	0,0097	0,0034	2,82	**0,0288**
										(MCI Aβ+) - (AD Aβ+)	0,0047	0,0031	1,50	0,4384
BA35	93,93	7,28	**1,9x10^{-4}**	0,20	122	CU Aβ–	-0,0021	-0,0045	0,0003	(CU Aβ–) - (CU Aβ+)	0,0006	0,0029	0,20	0,9972
						CU Aβ+	-0,0027	-0,0080	0,0026	(CU Aβ–) - (MCI Aβ+)	0,0075	0,0023	3,31	**0,0080**
						MCI Aβ+	-0,0097	-0,0135	-0,0058	(CU Aβ–) - (AD Aβ+)	0,0107	0,0029	3,75	**0,0015**
						AD Aβ+	-0,0128	-0,0179	-0,0077	(CU Aβ+) - (MCI Aβ+)	0,0070	0,0033	2,11	0,1603
										(CU Aβ+) - (AD Aβ+)	0,0101	0,0037	2,72	**0,0377**
										(MCI Aβ+) - (AD Aβ+)	0,0032	0,0032	0,98	0,7632
BA36	82,10	4,71	**4,4x10^{-3}**	0,17	111	CU Aβ–	-0,0016	-0,0038	0,0005	(CU Aβ–) - (CU Aβ+)	0,0015	0,0025	0,62	0,9260
						CU Aβ+	-0,0032	-0,0076	0,0013	(CU Aβ–) - (MCI Aβ+)	0,0049	0,0020	2,43	0,0807
						MCI Aβ+	-0,0065	-0,0099	-0,0031	(CU Aβ–) - (AD Aβ+)	0,0082	0,0025	3,27	**0,0076**
						AD Aβ+	-0,0098	-0,0143	-0,0053	(CU Aβ+) - (MCI Aβ+)	0,0034	0,0028	1,20	0,6309
										(CU Aβ+) - (AD Aβ+)	0,0067	0,0032	2,10	0,1600
										(MCI Aβ+) - (AD Aβ+)	0,0033	0,0028	1,17	0,6479
PHC	85,39	14,26	**1,3x10^{-7}**	0,19	124	CU Aβ–	-0,0018	-0,0035	-0,0002	(CU Aβ–) - (CU Aβ+)	0,0020	0,0020	1,00	0,7474
						CU Aβ+	-0,0038	-0,0075	-0,0002	(CU Aβ–) - (MCI Aβ+)	0,0070	0,0015	4,68	**0,0001**
						MCI Aβ+	-0,0088	-0,0113	-0,0063	(CU Aβ–) - (AD Aβ+)	0,0107	0,0020	5,37	**0,0000**
						AD Aβ+	-0,0125	-0,0161	-0,0089	(CU Aβ+) - (MCI Aβ+)	0,0050	0,0022	2,26	0,1186
										(CU Aβ+) - (AD Aβ+)	0,0087	0,0026	3,37	**0,0056**
										(MCI Aβ+) - (AD Aβ+)	0,0037	0,0022	1,67	0,3454

Supplementary Table 7. MTL subregions baseline volume among MCI-to-AD converters and non-converters. Statistic summary of the *group* main effect from multiple regressions, as the result of model testing with F test (sample size [n], F-values, degrees of freedom [df] and p-values are reported). Non-bolded p-values did not survive the Holm correction ($P<.05$). *BA, Brodmann area; ERC, entorhinal cortex; HPC, hippocampus (a, anterior; p, posterior); PHC, parahippocampal cortex; PRC, perirhinal cortex.*

Main effect: *group*					
	df	F	*P*	R^2	n
HPC	1	0,99	0,3244	0,08	52
aHPC	1	0,00	0,9966	-0,02	53
pHPC	1	4,43	0,0408	0,16	52
ERC	1	1,09	0,3026	0,08	52
PRC	1	7,30	0,0102	0,16	44
BA35	1	2,76	0,1036	-0,04	51
BA36	1	6,04	0,0185	0,21	46
PHC	1	1,44	0,2363	0,19	53

Supplementary Table 8. MTL subregions volume decline among MCI-to-AD converters and non-converters. Left: Statistic summary of the *group* x *time* interaction effect from LMMs, as the result of model testing with F test (sample size [n], F-values, approximate denominator degrees of freedom [ddf], p-values and marginal R^2 [i.e., approximation of the variance explained by the fixed effects] are reported). Bolded p-values survived the Holm correction (*P*<.05). Right: Estimated marginal trends of volume decline in each group (estimates and 95% confidence interval [CI] are reported). *BA, Brodmann area; ERC, entorhinal cortex; HPC, hippocampus (a, anterior; p, posterior); PHC, parahippocampal cortex; PRC, perirhinal cortex.*

	Interaction effect: *group* x *time*					Volume decline			
	ddf	F	*P*	R^2m	n	group	emtrend	95% CI	
HPC	56,14	15,09	**0,0003**	0,16	52	non-converter	-0,0107	-0,0135	-0,0078
						converter	-0,0205	-0,0247	-0,0163
aHPC	58,21	12,44	**0,0008**	0,08	53	non-converter	-0,0062	-0,0093	-0,0032
						converter	-0,0154	-0,0196	-0,0112
pHPC	56,13	9,28	**0,0035**	0,24	52	non-converter	-0,0103	-0,0129	-0,0077
						converter	-0,0172	-0,0209	-0,0134
ERC	57,62	2,69	0,1063	0,18	52	non-converter	-0,0093	-0,0139	-0,0046
						converter	-0,0157	-0,0221	-0,0094
PRC	41,26	2,23	0,1430	0,25	44	non-converter	-0,0073	-0,0104	-0,0043
						converter	-0,0109	-0,0146	-0,0072
BA35	53,27	0,04	0,8468	0,06	51	non-converter	-0,0083	-0,0115	-0,0051
						converter	-0,0088	-0,0133	-0,0044
BA36	44,57	2,75	0,1041	0,30	46	non-converter	-0,0043	-0,0080	-0,0007
						converter	-0,0090	-0,0132	-0,0047
PHC	58,14	4,10	0,0475	0,25	53	non-converter	-0,0061	-0,0081	-0,0041
						converter	-0,0096	-0,0123	-0,0068

Supplementary Table 9. Relationship between MTL-subregions volume and episodic memory/executive functions/speed processing performance in patients of the Alzheimer's continuum. Statistic summary of the *volume* main effect from LMMs (sample size [n], estimates, standard errors [SE], degree of freedom [df], t-ratios and p-values are reported). Bolded p-values survived the Holm correction (P<.05). *BA, Brodmann area; ERC, entorhinal cortex; HPC, hippocampus (a, anterior; p, posterior); PHC, parahippocampal cortex; PRC, perirhinal cortex.*

	cognitive score	estimate	SE	df	t	*P*	n
HPC	episodic memory	0,169	0,058	37,29	2,92	**0,0060**	43
	executive functions	-0,045	0,094	34,59	-0,48	0,636	40
	speed processing	-0,164	0,231	45,00	-0,71	0,4798	58
aHPC	episodic memory	0,134	0,062	39,18	2,16	0,0373	44
	executive functions	0,007	0,092	34,24	0,08	0,938	41
	speed processing	-0,201	0,234	43,13	-0,86	0,3956	59
pHPC	episodic memory	0,207	0,075	38,65	2,75	**0,0091**	43
	executive functions	-0,122	0,120	37,44	-1,02	0,314	40
	speed processing	-0,119	0,291	50,91	-0,41	0,6845	58
ERC	episodic memory	0,218	0,069	42,92	3,14	**0,0030**	44
	executive functions	-0,081	0,107	41,79	-0,75	0,457	41
	speed processing	-0,155	0,260	53,60	-0,59	0,5545	59
PRC	episodic memory	0,198	0,068	35,27	2,88	**0,0066**	39
	executive functions	0,008	0,125	29,66	0,07	0,947	36
	speed processing	-0,142	0,266	42,29	-0,53	0,5964	51
BA35	episodic memory	0,176	0,063	35,73	2,80	**0,0082**	42
	executive functions	-0,097	0,111	31,41	-0,88	0,387	39
	speed processing	-0,062	0,262	43,59	-0,24	0,8131	57
BA36	episodic memory	0,186	0,079	37,85	2,35	0,0244	40
	executive functions	0,094	0,133	31,35	0,71	0,485	37
	speed processing	-0,137	0,285	45,93	-0,48	0,6326	52
PHC	episodic memory	0,101	0,103	40,70	0,98	0,3346	44
	executive functions	-0,169	0,157	37,86	-1,08	0,289	41
	speed processing	0,588	0,367	48,92	1,60	0,1154	59

2.2 Supplementary Figures

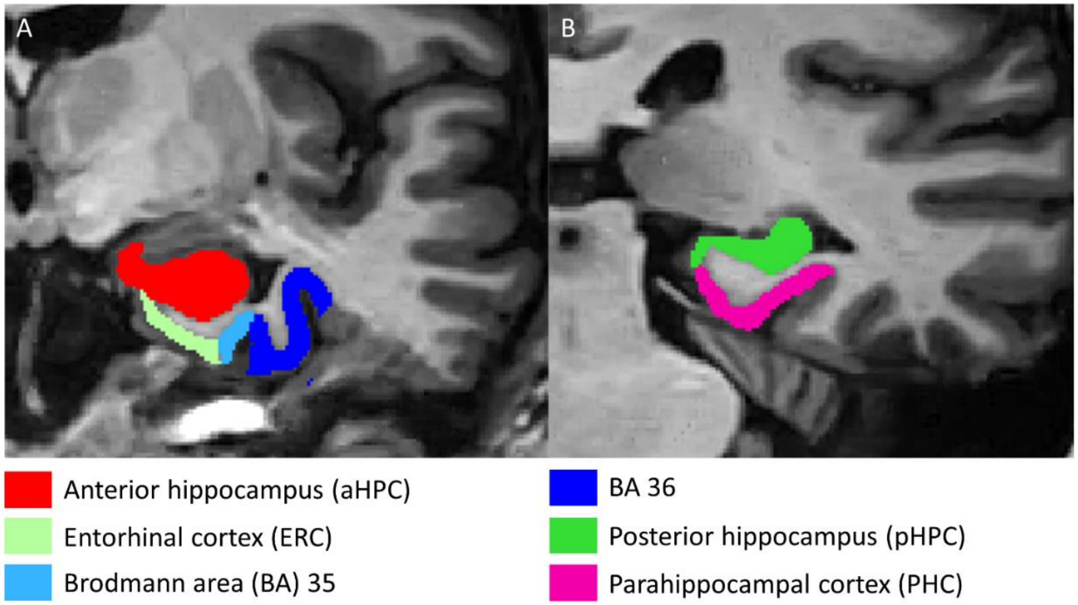

Supplementary Figure 1. Segmentation of MTL subregions using ASHS-T1 (Xie et al., 2019). An anterior (A) and posterior (B) slice from the same subject are displayed.

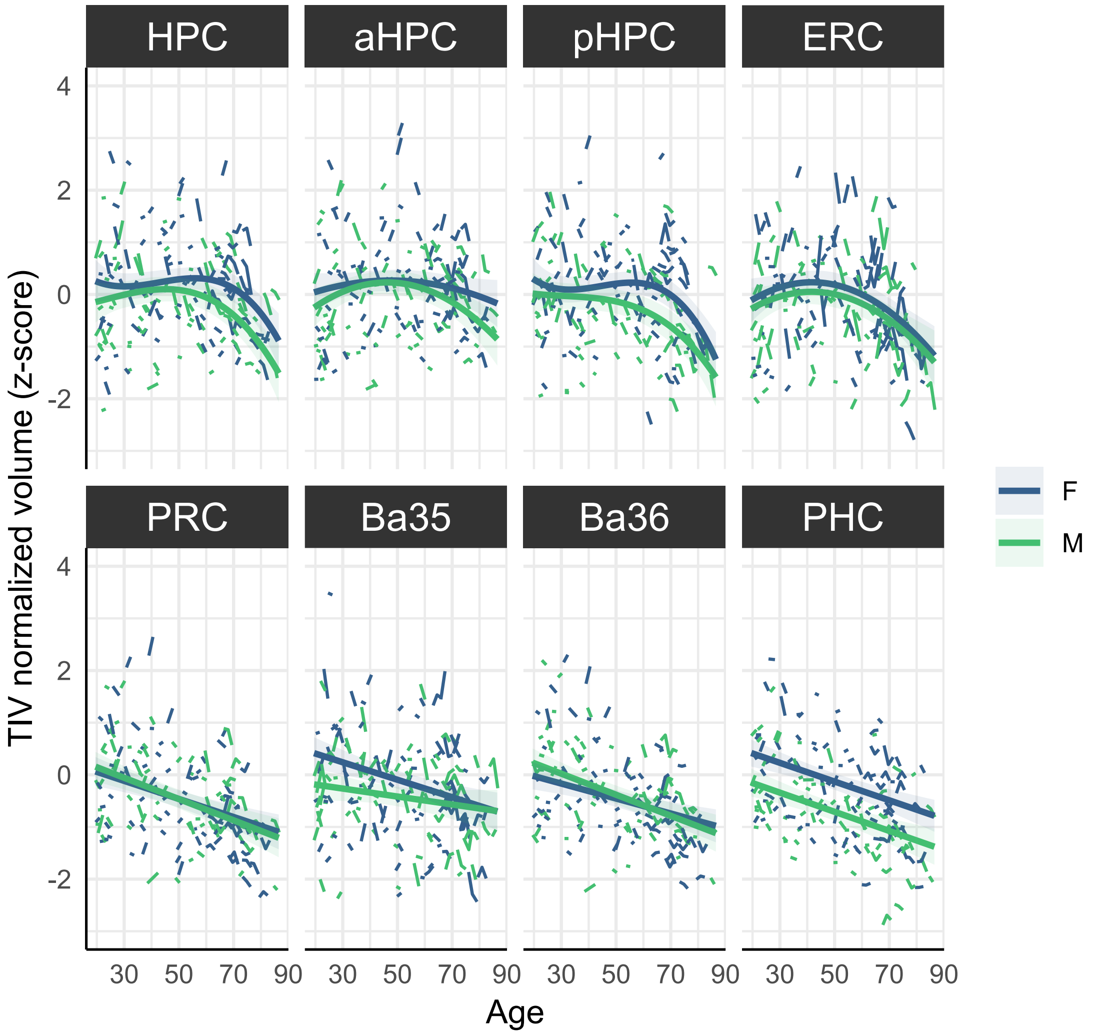

Supplementary Figure 2. MTL subregions age-related volume trajectories across the adult lifespan in female and male. No significant interaction *age* x *sex* was found in none of all models but a main effect of *sex* appears significant for the pHPC and PHC, suggesting that females had greater volumes adjusted for the total intracranial volume (*P*<.05). Continuous line indicates model-derived estimates. Shade area represents 95% confidence interval. *BA, Brodmann area; ERC, Entorhinal cortex; HPC, Hippocampus (a, anterior; p, posterior); PHC, Parahippocampal cortex; PRC, Perirhinal cortex.*
